# Supplementary material for: Nitroglycerin-responsive gene switch for the on-demand production of therapeutic proteins
Source: Nat Biomed Eng. 2025 Feb 14;9(7):1129–43. doi: 10.1038/s41551-025-01350-7 (PMC12270905; doi:10.1038/s41551-025-01350-7)
Supplement: Supplementary file 1 — Supplementary figures, tables and references. [file 41551_2025_1350_MOESM1_ESM.pdf]

nature biomedical engineering

Supplementary information https://doi.org/10.1038/s41551-025-01350-7

Nitroglycerin-responsive gene switch for the on-demand production of therapeutic proteins

In the format provided by the authors and unedited

Table of Contents

Supplementary Fig.1 1

Supplementary Fig.2 2

Supplementary Fig.3..3

Supplementary Fig.4..4

Supplementary Fig.5..6

Supplementary Fig.6..7

Supplementary Table 1..8

Supplementary Table 2..11

Supplementary Table 3..11

Supplementary References...15

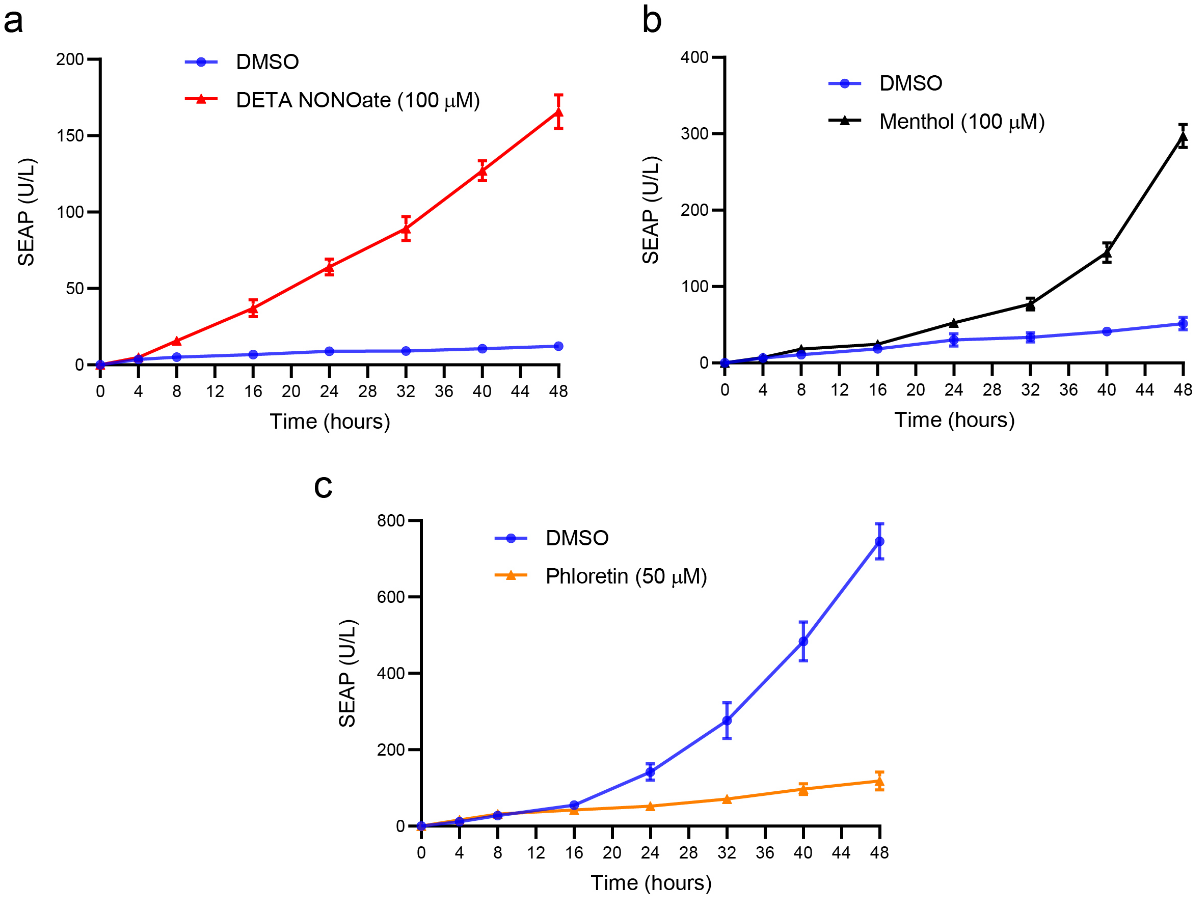

Supplementary Fig. 1 | Expression kinetics of hNORM compared with menthol- and phloretin-inducible gene expression systems in mammalian cells. HEK-293 cells were co-transfected either with **(a)** pMMH178 (P_{hPGK}-sGCα-pA_{bGH}), pMMH179 (P_{hPGK}-sGCβ-pA_{bGH}), PKG1β WT (P_{hCMV}-PKG1β-pA_{bGH}), and pCK53 (P_{CRE}-SEAP-pA_{bGH}), **(b)** phTRPM8 (P_{hCMV}-hTRPM8-pA_{bGH}) and pMX57 (P_{NFAT3}-SEAP- pA_{bGH}), or **(c)** pMG11 (P_{SV40}-TtgA1- pA_{bGH}) and pMG10 (P_{TtgR1}-SEAP- pA_{bGH}). At 24 h following the transfection, the medium was changed to 100 µl of fresh medium containing either DMSO, DETA NONOate (100 µM), menthol (100 µM), or phloretin (20 µM), respectively. Samples from the supernatants were taken every 4 h, starting immediately after the drug addition. Data are presented as means ± s.d., n = 3. Source data are provided as a Source Data file.

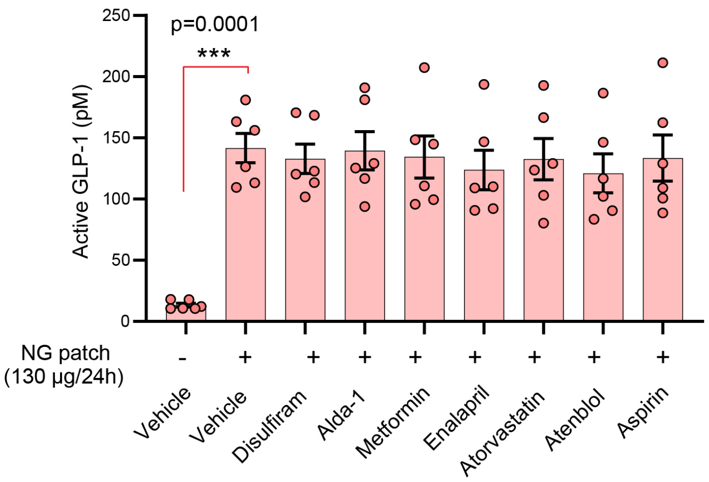

Supplementary Fig. 2 | hNORM performance in mice is not affected by co-administration of ALDH2 inhibitor/inducer or commonly prescribed drugs. Wild-type C57BL/6 mice were randomly divided into nine groups and transplanted with microencapsulated HEK_{hNORM3} cells ($5x10^6$ cells per mouse). At 24 h after implantation, NG patches (130 µg/24 h) were transdermally placed on top of the implant area along with intraperitoneal administration of vehicle, disulfiram (100 mg/kg), Alda-1 (2 mg/kg), metformin (125 mg/kg), enalapril (30 mg/kg), atorvastatin (40 mg/kg), bisoprolol (10 mg/kg), or aspirin (50 mg/kg). Mice untreated with NG patches served as a negative control. At 24 h following drug treatment, blood samples were taken, and serum GLP-1 levels were quantified. Data are presented as mean ± SEM, n = 6. Statistical significance was analyzed by two-way ANOVA and p values were calculated using Tukey`s multi-comparison tests. Source data are provided as a Source Data file.

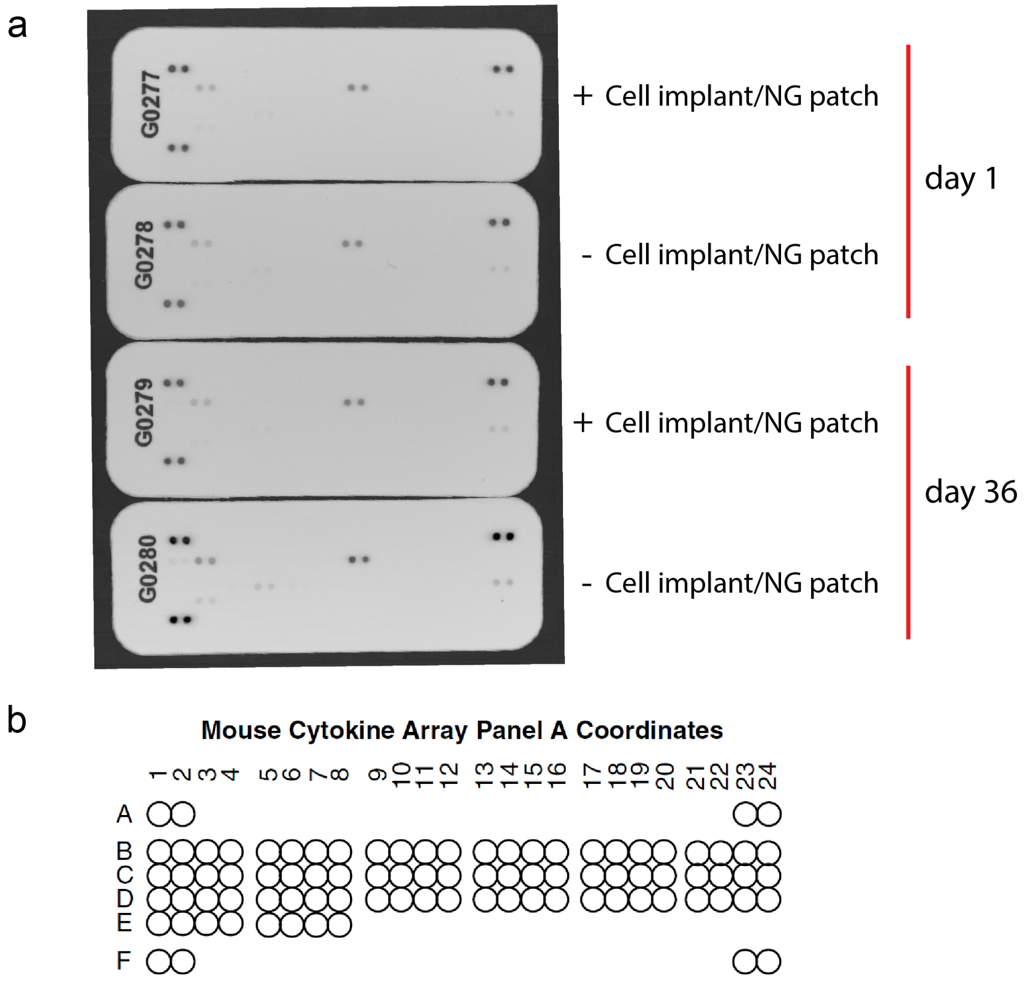

Refer to the table below for the Mouse Cytokine Array coordinates.

Coordinate	Target/Control	Alternate Nomenclature
A1, A2	Reference Spot	—
A23, A24	Reference Spot	—
B1, B2	BLC	CXCL13/BCA-1
B3, B4	C5/C5a	Complement Component 5a
B5, B6	G-CSF	—
B7, B8	GM-CSF	—
B9, B10	I-309	CCL1/TCA-3
B11, B12	Eotaxin	CCL11
B13, B14	ICAM-1	CD54
B15, B16	IFN-γ	—
B17, B18	IL-1α	IL-1F1
B19, B20	IL-1β	IL-1F2
B21, B22	IL-1ra	IL-1F3
B23, B24	IL-2	—
C1, C2	IL-3	—
C3, C4	IL-4	—
C5, C6	IL-5	—
C7, C8	IL-6	—
C9, C10	IL-7	—
C11, C12	IL-10	—
C13, C14	IL-13	—
C15, C16	IL-12 p70	—
C17, C18	IL-16	—
C19, C20	IL-17	—
C21, C22	IL-23	—
C23, C24	IL-27	—

Coordinate	Target/Control	Alternate Nomenclature
D1, D2	IP-10	CXCL10/CRG-2
D3, D4	I-TAC	CXCL11
D5, D6	KC	CXCL1
D7, D8	M-CSF	—
D9, D10	JE	CCL2/MCP-1
D11, D12	MCP-5	CCL12
D13, D14	MIG	CXCL9
D15, D16	MIP-1α	CCL3
D17, D18	MIP-1β	CCL4
D19, D20	MIP-2	CXCL2
D21, D22	RANTES	CCL5
D23, D24	SDF-1	CXCL12
E1, E2	TARC	CCL17
E3, E4	TIMP-1	—
E5, E6	TNF-α	—
E7, E8	TREM-1	—
F1, F2	Reference Spot	—
F23, F24	PBS (Negative Control)	Control (-)

Supplementary Fig. 3 | Serum cytokines profiling at the beginning and after 35 days of treatment with hNORM-regulated GLP-1. (a) Quantitative immunoblotting of mouse cytokines in serum at day 1 and day 36 as described in the experiment shown in **Fig.6. (b)** Description of spot coordinates presented in **a**.

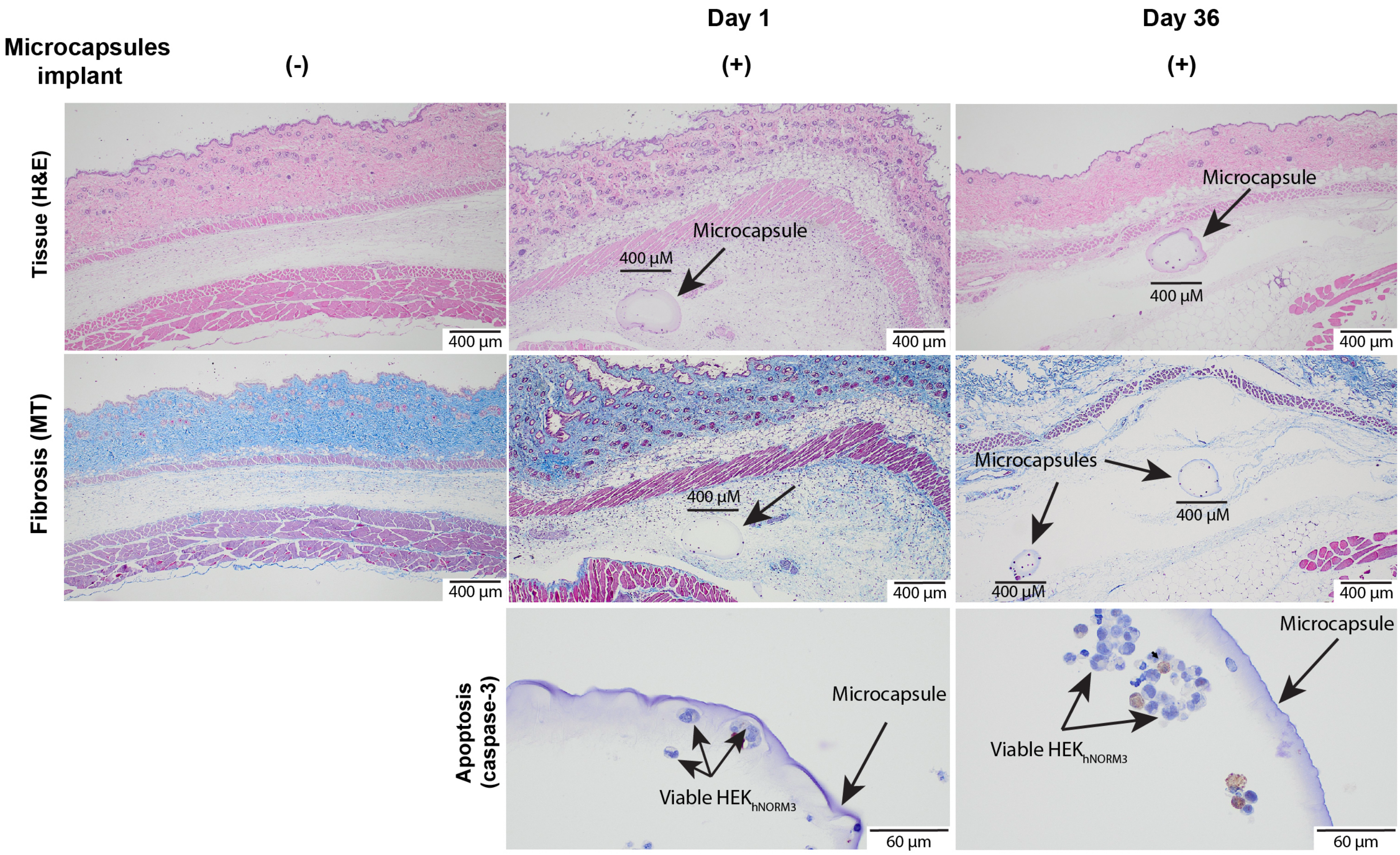

Supplementary Fig. 4 | Histological analysis of subcutaneous implants at the beginning and at the end of 35 days of hNORM treatment. Hematoxylin and eosin (H&E) staining, Masson's trichrome (MT) staining, and anti-caspase-3 immunohistochemistry of encapsulated cells subcutaneously implanted in mice at day 1 and day 36 as described in the experiment shown in **Fig. 6**. Black arrows point to implanted microcapsules (average diameter: 400 µm). Fibrosis surrounding the microcapsules and apoptosis inside the implant are insignificant in MT staining and IHC using apoptosis marker caspase 3, respectively. Diagnostic criteria used throughout the study are based on recognized texts and current scientific literature, as well as ISO 19001:2013.

a

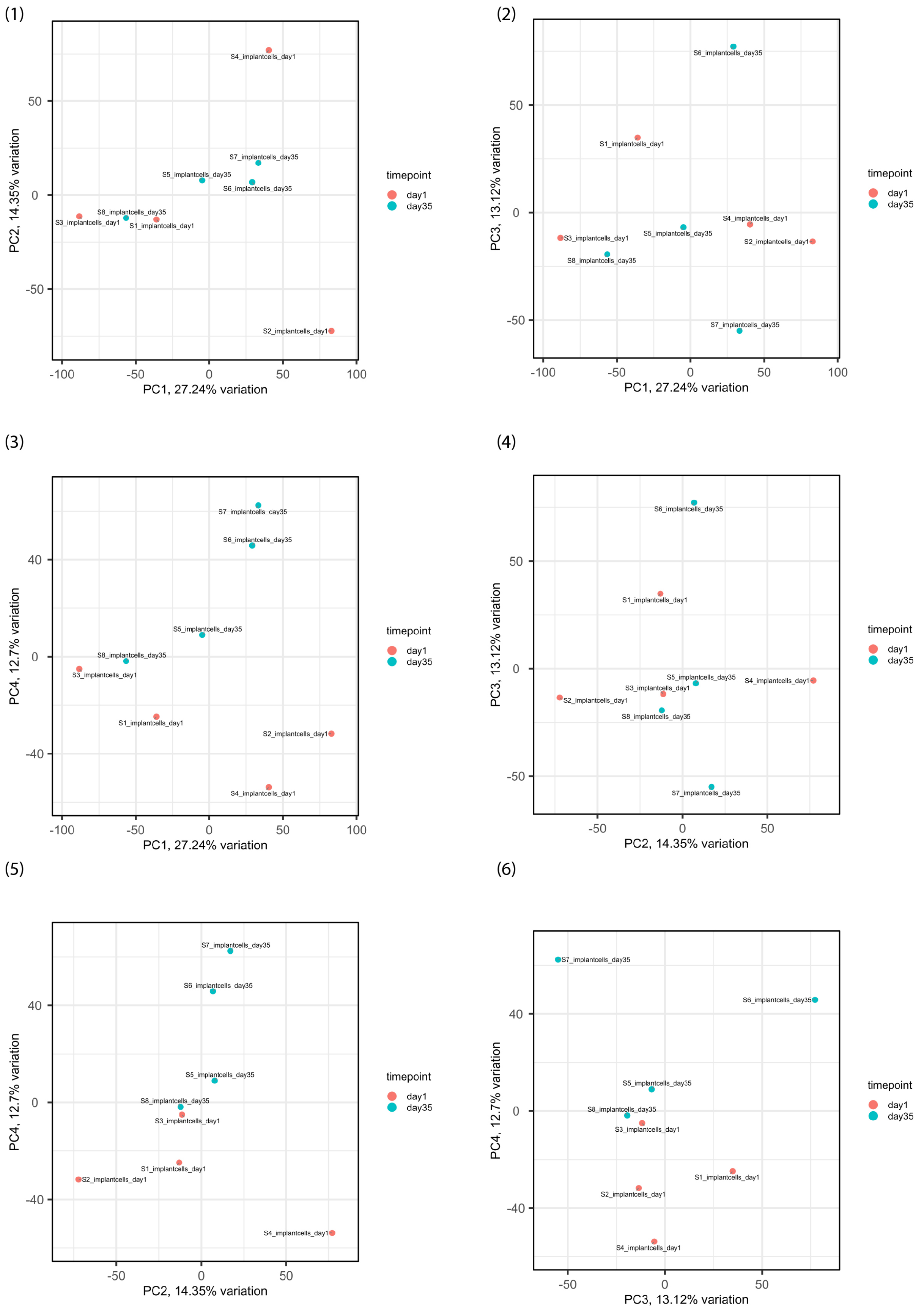
(1)
PC1, 27.24% variation
PC2, 14.35% variation
S4_implantcells_day1
S7_implantcells_day35
S5_implantcells_day35
S6_implantcells_day35
S8_implantcells_day35
S3_implantcells_day1
S1_implantcells_day1
S2_implantcells_day1
timepoint
day1
day35
(2)
PC1, 27.24% variation
PC3, 13.12% variation
S6_implantcells_day35
S1_implantcells_day1
S4_implantcells_day1
S5_implantcells_day35
S3_implantcells_day1
S2_implantcells_day1
S8_implantcells_day35
S7_implantcells_day35
timepoint
day1
day35
(3)
PC1, 27.24% variation
PC4, 12.7% variation
S7_implantcells_day35
S6_implantcells_day35
S5_implantcells_day35
S8_implantcells_day35
S3_implantcells_day1
S1_implantcells_day1
S2_implantcells_day1
S4_implantcells_day1
timepoint
day1
day35
(4)
PC2, 14.35% variation
PC3, 13.12% variation
S6_implantcells_day35
S1_implantcells_day1
S5_implantcells_day35
S4_implantcells_day1
S3_implantcells_day1
S2_implantcells_day1
S8_implantcells_day35
S7_implantcells_day35
timepoint
day1
day35
(5)
PC2, 14.35% variation
PC4, 12.7% variation
S7_implantcells_day35
S6_implantcells_day35
S5_implantcells_day35
S8_implantcells_day35
S3_implantcells_day1
S1_implantcells_day1
S2_implantcells_day1
S4_implantcells_day1
timepoint
day1
day35
(6)
PC3, 13.12% variation
PC4, 12.7% variation
S7_implantcells_day35
S6_implantcells_day35
S5_implantcells_day35
S8_implantcells_day35
S3_implantcells_day1
S1_implantcells_day1
S2_implantcells_day1
S4_implantcells_day1
timepoint
day1
day35

b

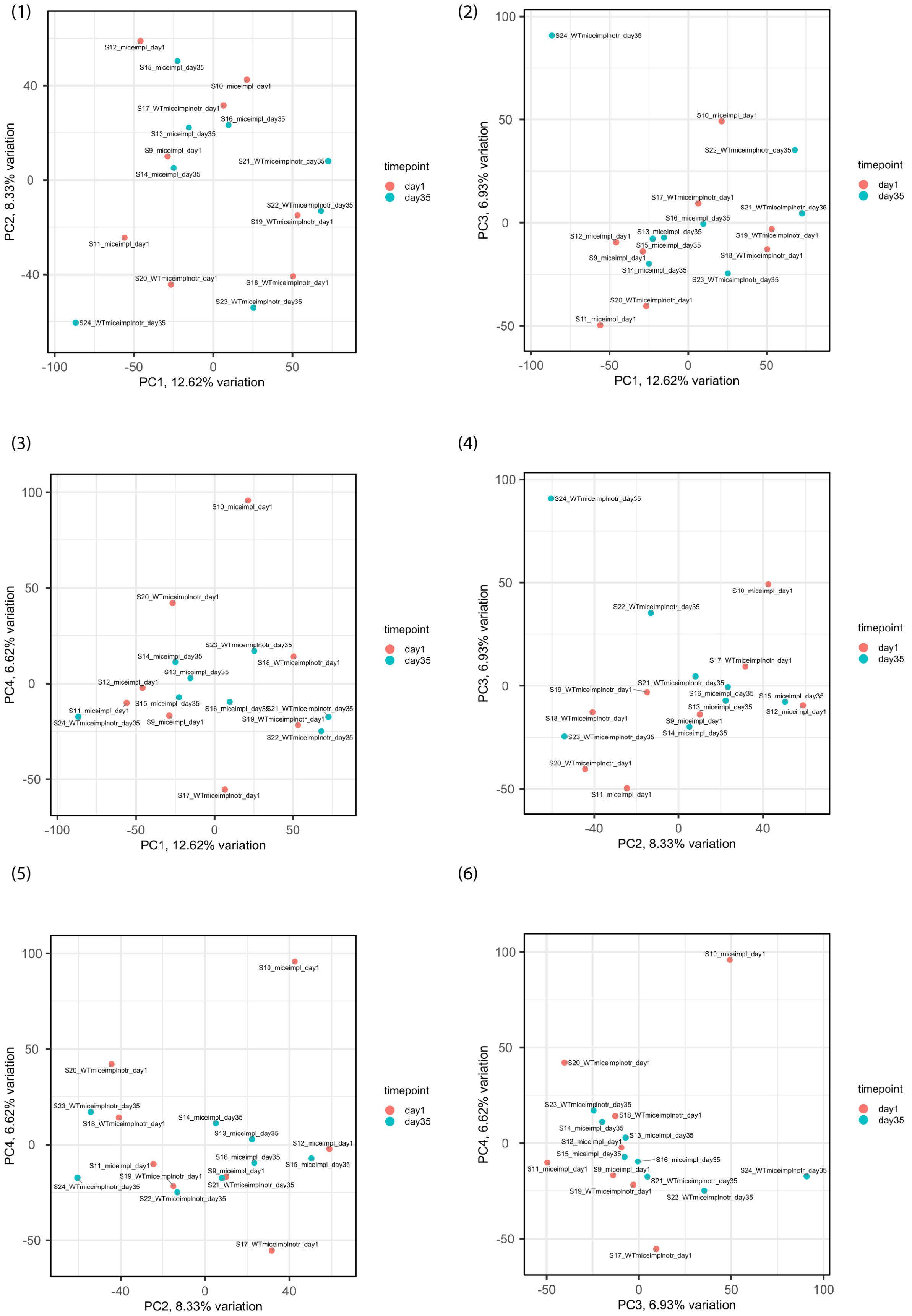

Supplementary Fig. 5 | PCA analysis of RNAseq samples obtained at the beginning and the end of 35 days of hNORM treatment. (a) PCA analysis of encapsulated cells at day 1 and day 35 after implantation and hNORM treatment as described in the experiment shown in **Fig. 6**. The most informative dimensions do not separate the day 1 and day 35 samples and edgeR analysis revealed no statistical significance in differential expression of genes. **(b)**The same PCA analysis as in (a) for mouse tissues surrounding the implants. There is no obvious separation between samples on day 1 and day 35 in PCA dimensions 1-4 plotting for either the engineered cells or the mouse tissues.

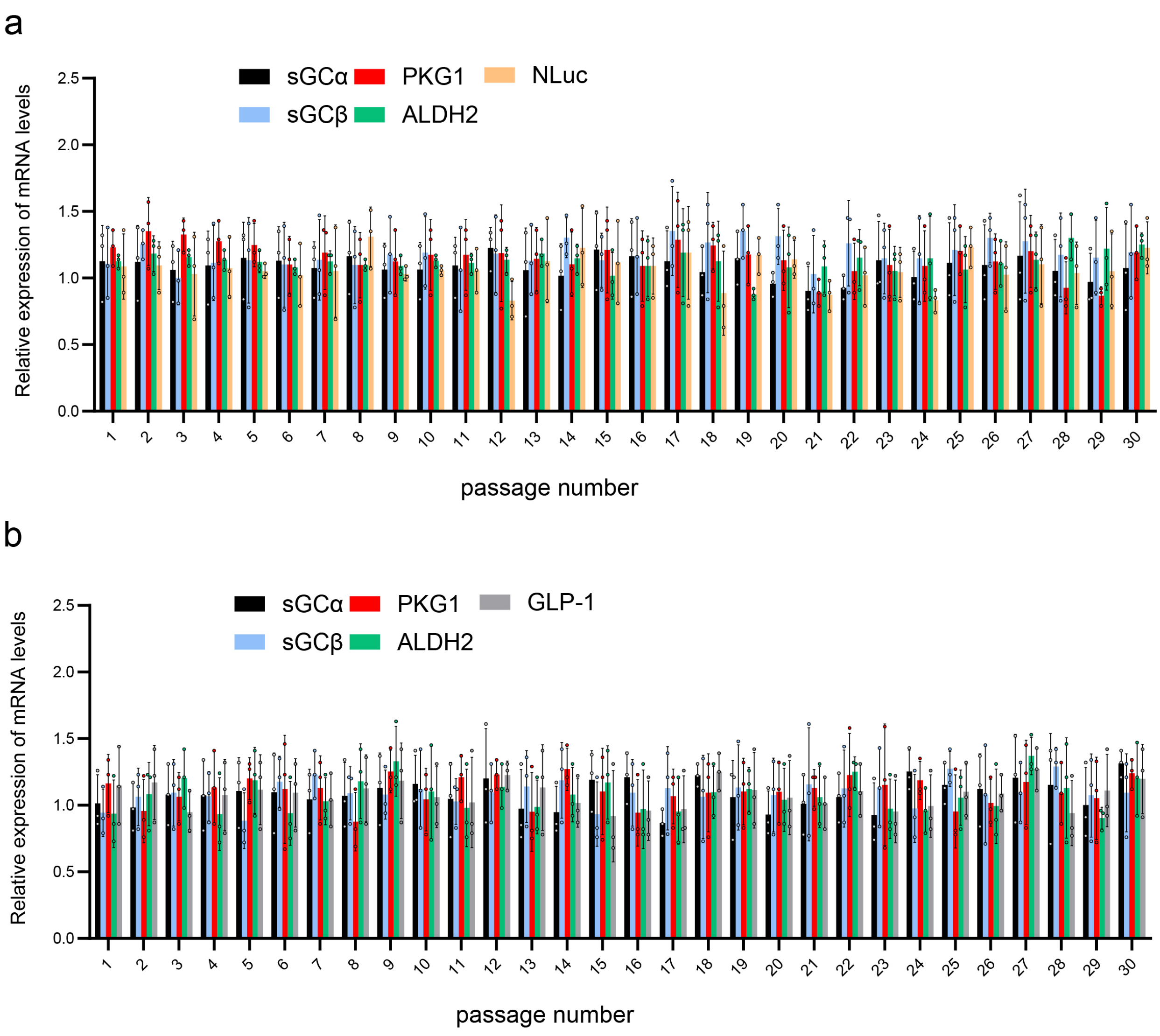

Supplementary Fig.6 | Stability assessment of HEK_{hNORM2} and HEK_{hNORM3} cell lines performed by qPCR analysis of introduced transgenes. Transgene mRNA levels of **(a)** HEK_{hNORM2} and **(b)** HEK_{hNORM3} cell lines were quantified by qPCR for 30 consecutive passages. Data are presented as means ± s.d., n = 3. Statistical significance was analyzed by two-way ANOVA. Source data are provided as a Source Data file.

Supplementary Table 1

Plasmid	Information and Design	Reference
pcDNA3.1(+)	Mammalian expression vector.<br>(P_{hCMV}-MCS-pA_{bGH})	Life Technologies, Carlsbad, CA
ALDH2	A plasmid that contains human ALHD2 coding sequence (DNASU plasmid no. HsCD00003248). This plasmid was used as a PCR template for cloning ALHD2 in a mammalian expression vector.	DNASU Plasmid Repository
pTS395	Mammalian expression vector encodes Sleeping Beauty transposase (SBx100).<br>(P_{hCMV}-SBx100-pA_{bGH})	[1]
pVAMSyB Tier1	Tier1 mammalian expression vector.<br>(P_{hCMV}-MCS-pA_{bGH})	[1]
Tier3(SB)-YPet-Puro	Tier3 vector for stable integration via Sleeping Beauty transposase (SB) which includes a YPet marker and Puro resistance gene.<br>(SB_{ITR}-MCS-pA_{bGH}: P_{hCMV}-YPet-p2A-Puro-pA_9-SB_{ITR})	[1]
Tier3(SB)-mTagBFP2-Blast	Tier3 vector for stable integration via Sleeping Beauty transposase (SB) which includes a mTagBFP2 marker and Blast resistance gene.<br>(SB_{ITR}-MCS-pA_{bGH}: P_{hCMV}-mTagBFP2-p2A-Blast-pA_9-SB_{ITR})	[1]
Tier3(SB)-mRuby2-Zeo	Tier3 vector for stable integration via Sleeping Beauty transposase (SB) which includes a mRuby2 marker and Zeo resistance gene.<br>(SB_{ITR}-MCS-pA_{bGH}: P_{hCMV}-mRuby2-p2A-Zeo-pA_9-SB_{ITR})	[1]
Tier3(SB)-iRFP670-Hygro	Tier3 vector for stable integration via Sleeping Beauty transposase (SB) which includes an iRFP670 marker and Hygro resistance gene.<br>(SB_{ITR}-MCS-pA_{bGH}: P_{hCMV}-iRFP670-p2A-Hygro-pA_9-SB_{ITR})	[1]
pMMH37	Constitutive mammalian expression vector encodes SS-FRB-ssTEV$p_{119\text{-}245}$-KDEL.<br>(P_{hPGK}-SS-FRB-ssTEV$p_{119\text{-}245}$-KDEL-pA_{bGH})	[2]
pGLP1R	Mammalian expression plasmid encodes GLP-1 receptor (GLP1R).<br>(P_{hCMV}-GLP1R-pA_{bGH})	[3]
PKGIβ WT	Mammalian expression vector encodes human PKGIβ under the regulation of CMV promoter (addgene no.16394).<br>(P_{hCMV}-PKG1β-pA_{bGH})	[4]
PRKG2	Mammalian expression vector encodes human PKGII under the regulation of CMV promoter (addgene no.23435).<br>(P_{hCMV}-PKG2-pA_{bGH})	[5]
pCK53	Mammalian expression vector encodes SEAP reporter gene under the regulation of CRE promoter.<br>(P_{CRE}-SEAP-pA_{bGH})	[6]
phTRPM8	Mammalian expression vector encodes human TRPM8 under the regulation of CMV promoter.<br>(P_{hCMV}-hTRPM8-pA_{bGH})	[7]
pJH532	Mammalian expression vector encodes GLP-1-FC-P2A-NLuc under the regulation of CRE promoter.<br>(P_{CRE}-GLP-1-FC-P2A-NLuc-pA_{bGH})	[8]
pMG10	Mammalian expression vector encodes human SEAP under the regulation of TtgR1 promoter.<br>(P_{TtgR1}-SEAP- pA_{bGH})	[9]
pMG11	Mammalian expression vector encodes human TtgA1 under the regulation of SV40 promoter.<br>(P_{SV40}-TtgA1- pA_{bGH})	[9]

pMX57	Mammalian expression vector encodes SEAP reporter gene under the regulation of NFAT3 promoter.<br>(P_{NFAT3}-SEAP- pA_{bGH})	[10]
pBD067	Mammalian expression vector encodes TurboGFP under the regulation of CRE promoter in pVAMSyB Tier1.<br>(P_{CRE}-TruboGFP-$pA_{bGH)}$	Danuser et al., unpublished
pBD469	Mammalian expression vector encodes iRFP670 under the regulation of CMV promoter in pVAMSyB Tier1.<br>(P_{hCMV}-iRFP670-$pA_{bGH)}$	Danuser et al., unpublished
pMMH83	Mammalian expression vector encodes IgK_{SS}-NLuc-3xFLAG. The DNA which encodes IgK_{SS}-NLuc-3xFLAG was synthesized by TWIST and cloned into pVAMSyB Tier1 through *EcoR*I/*Hin*dIII restriction enzymes.<br>(P_{hCMV}-NLuc-3xFLAG-pA_{bGH})	This work
pMMH178	Mammalian expression vector encodes the α chain of human sGC 1 (GUCY1A1). The DNA encoding sequence was synthesized by TWIST and cloned into pMMH37 through *Eco*RI/*Sbf*I restriction enzymes.<br>(P_{hPGK}-sGCα-pA_{bGH})	This work
pMMH179	Mammalian expression vector encodes the β chain of human sGC (GUCY1B1). The DNA encoding sequence was synthesized by TWIST and cloned into pMMH37 through *Eco*RI/*Sbf*I restriction enzymes.<br>(P_{hPGK}-sGCβ-pA_{bGH})	This work
pMMH185	Mammalian expression vector encodes the α and the β chains of human sGC for stable integration. PGK-αsGC was PCR amplified from pMMH178 using the following primers: 5`-taagagatctacgcgtctaccgggtaggggaggcgcttttcccaag and 5`-gtcaccagcctgcttcagcaggctgaagttagtagctccgcttccaccagaaccaccactatctattcctgatgctttgcctaaaaaatt. βsGC was PCR amplified from pMMH179 using the following primers: 5`-ggagctactaacttcagcctgctgaagcaggctggtgacgtggaggagaaccctggacctatgtacggatttgtgaatcacgccctggag and 5`-gccggcctcaaagcttctagtcatcatcctgctttgtttcctctgt.<br>The resulting PCR products were cloned into *Mlu*I/*Hin*dIII digested Tier3(SB)- iRFP670-Hygro through Gibson assembly.<br>(SB_{ITR}-P_{hPGK}-sGCα-P2A-sGCβ-pA_{bGH}: P_{SV40}-iRFP670-P2A-Hygro-pA_{p9}-SB_{ITR})	This work
pMMH186	Mammalian expression vector encodes NLuc under the regulation of CRE promoter. CRE promoter was PCR amplified from pCK53 using the following primers: 5`-taagagatctacgcgtgcaccagacagtgacgtcagctgccagatc and 5`-tctcactagtcatggtgcttcatccccgtggcccgttgctcgcgtt.<br>NLuc was PCR amplified from pMMH83 using the following primers: 5`- accatgactagtgagacagacacactcctgcta and 5`-ggccggcctcaaagcttctatcgcaggccccaggacacgcctatag<br>The resulting PCR products were cloned into *Mlu*I/*Hin*dIII digested Tier3(SB)-YPet-Puro through Gibson assembly.<br>(SB_{ITR}-P_{CRE}-NLuc-pA_{bGH}: P_{hCMV}-YPet-P2A-Puro-pA_{p9}-SB_{ITR})	This work

pMMH187	Mammalian expression vector encodes human ALDH2 under CMV promoter. CMV promoter was PCR-amplified from pVAMSyB Tier1 using the following primers: 5`-taagagatctacgcgttagttattaatagtaatcaattacggggtc and 5`-cggcagcgcgcaacatgctagccagcttgggtctccctatagtgagtcgtatta.<br>Human ALHD2 was PCR amplified from a plasmid that contains ALHD2 encoding sequence (DNASU plasmid no. HsCD00003248) using the following primers:<br>5`- atgttgcgcgctgccgcccgcttcgggccc and 5`-gccggcctcaaagctttctagacaccggtggatccgctagcactag.<br>The resulting PCR products were cloned into *Mlu*I/*Hin*dIII digested Tier3(SB)-mTagBFP2-Blast through Gibson assembly.<br>(SB$_{ITR}$-P$_{hCMV}$-ALDH2-pA$_{bGH}$: P$_{hCMV}$-mTagBFP2-P2A-Blast-pA$_{p9}$-SB$_{ITR}$)	This work
pMMH188	Mammalian expression vector encodes human PGK1 β under CMV promoter. PGK1 expression cassette was PCR amplified from PKGIβ WT (addgene no.16394) using the following primers:<br>5`-taagagatctacgcgttagttattaatagtaatcaattacggggtc and 5`-gccggcctcaaagctttctagacaccggtggatccgctagcactag<br>The resulting PCR products were cloned into *Mlu*I/*Hin*dIII digested Tier3(SB)-mRuby2-Zeo through Gibson assembly.<br>(SB$_{ITR}$-P$_{hCMV}$-PGK1-pA$_{bGH}$: P$_{hCMV}$-mRuby2-P2A-Zeo-pA$_{p9}$-SB$_{ITR}$)	This work
pMMH213	Mammalian expression vector encodes GLP-1-Fc-P2A-NLuc under the regulation of CRE promoter. CRE promoter was PCR amplified from pCK53 using the following primers: 5`-taagagatctacgcgtgcaccagacagtgacgtcagctgccagatc and 5`-cacaggatgatcttactagtcatggtgcttcatccccgtggcccgttgctcgcgtt<br>GLP-1-Fc-P2A-NLuc was PCR amplified from pJH532 using the following primers: 5`-atgactagtaagatcatcctgtggctgtgt and 5`-gccggcctcaaagctttctagacaccggtggatccctacgccagaatgcgttcgcacagccg<br>The resulting PCR products were cloned into *Mlu*I/*Hin*dIII digested Tier3(SB)-YPet-Puro through Gibson assembly.<br>(SB$_{ITR}$-P$_{CRE}$-GLP-1-Fc-P2A-NLuc-pA$_{bGH}$: P$_{hCMV}$-YPet-P2A-Puro-pA$_{p9}$-SB$_{ITR}$)	This work

Abbreviations: **ALDH2,** Aldehyde dehydrogenase 2; **Blast,** blasticidin; **CRE,** cAMP response element, **Fc,** fragment crystallizable region; **FRB**, FKBP-rapamycin binding; **GFP**, green fluorescent protein; **GLP-1**, glucagon-like peptide-1; **GLP1R**, glucagon-like peptide-1 receptor; **Hygro,** hygromycin; **IgK,** immunoglobulin kappa; **iRFP670,** near-infrared fluorescent protein; **KDEL**, lysine, aspartic acid, glutamic acid, leucine; **mRuby2,** a red fluorescent protein derived from *Entacmaea quadricolor*; **mTagBFP,** a blue fluorescent protein derived from *Entacmaea quadricolor*. **SB$_{ITR}$**, inverted terminal repeats of Sleeping Beauty (SB) transposase; **MCS**, multiple cloning site; **NLuc**, nanoluciferase reporter gene; **P2A,** picornavirus-derived ribosome skipping sequence optimized for bicistronic expression in mammalian cells; **pA$_{bGH}$**, polyadenylation signal from the bovine growth hormone; **pA$_{p9}$**, synthetic polyadenylation signals; **PCR**, polymerase chain reaction; **P$_{hCMV}$**, human cytomegalovirus immediate early promoter; **P$_{hPGK}$**, human 3-phosphoglycerate kinase promoter; **P$_{SV40}$,** early promoter of the simian virus 40; **PGK,** protein kinase G; **Puro,** puromycin; **TEVp**, tobacco etch virus protease, **SB,** Sleeping Beauty transposase; **SEAP**, human placental secreted alkaline phosphatase; **sGC,** soluble guanylyl cyclase; **SS,** secretion signal; **YPet,** a yellow fluorescent protein, derived from *Aequorea victoria*; **Zeo,** zeocin; **PKGIβ,** cGMP-dependent protein kinase Iβ.

Supplementary Table 2

Gene	Primers
ALDH2	Forward 5`-ccaacaattccacgtacgggctgg<br>Reverse 5`- ggctccaaacacatcatagcagttgaccc
KGIβ	Forward 5`- cctgactggcagcccacctttct<br>Reverse 5`- cccagttaaagccctcaaaccatttgtgc
sGCα	Forward 5`- gaacaagcccgagctcaagatggc<br>Reverse 5`- gcaacctcacagggaaatatggagcac
sGCβ	Forward 5`- caagcatgcatctggagaaggagcc<br>Reverse 5`- tgtcatatacttgtcaccaacagtctccacc
GAPDH	Forward 5`- gtctcctctgacttcaacagcg<br>Reverse 5`- accaccctgttgctgtagccaa
GLP1	Forward 5`- gcggcagatctggttgtaagcctt<br>Reverse 5`- gtgtgcacctccacatcatctacaaacc
NLuc	Forward 5`- gctacaacctggaccaagtccttgaac<br>Reverse 5`- catttggtcgccgctcagacctt

Abbreviations: **ALDH2,** aldehyde dehydrogenase 2; **PKGIβ,** cGMP-dependent protein kinase Iβ; **sGC,** soluble guanylyl cyclase; **GAPDH,** glyceraldehyde 3-phosphate dehydrogenase; **GLP-1**, glucagon-like peptide-1; **NLuc**, nanoluciferase reporter gene.

Supplementary Table 3

Gene	Sequence
NLuc-3xFLAG	atgactagtgagacagacacactcctgctatgggtactgctgctctgggttccaggttccactggtgacgctag<br>tggtggttctggtatggtcttcacactcgaagatttcgttggggactggcgacagacagccggctacaacctgg<br>accaagtccttgaacagggaggtgtgtccagtttgtttcagaatctcggggtgtccgtaactccgatccaaagg<br>attgtcctgagcggtgaaaatgggctgaagatcgacatccatgtcatcatcccgtatgaaggtctgagcggcg<br>accaaatgggccagatcgaaaaaatttttaaggtggtgtaccctgtggatgatcatcactttaaggtgatcctgc<br>actatggcacactggtaatcgacggggttacgccgaacatgatcgactatttcggacggccgtatgaaggcat<br>cgccgtgttcgacggcaaaaagatcactgtaacagggaccctgtggaacggcaacaaaattatcgacgagc<br>gcctgatcaaccccgacggctccctgctgttccgagtaaccatcaacggagtgaccggctggcggctgtgcg<br>aacgcattctggcggactacaaagaccatgacggtgattataaagatcatgacatcgattacaaggatgacga<br>tgacaagtag
guanylate cyclase 1<br>soluble subunit<br>alpha 1 (GUCY1A1)	atgttctgcacgaagctcaaggatctcaagatcacaggagagtgtcctttctccttactggcaccaggtcaagtt<br>cctaacgagtcttcagaggaggcagcaggaagctcagagagctgcaaagcaaccgtgcccatctgtcaaga<br>cattcctgagaagaacatacaagaaagtcttcctcaaagaaaaaccagtcggagccgagtctatcttcacactt<br>tggcagagagtatttgcaaactgattttcccagagtttgaacggctgaatgttgcacttcagagaacattggcaa<br>agcacaaaataaaagaaagcaggaaatctttggaaagagaagactttgaaaaaacaattgcagagcaagca<br>gttgcagcaggagttccagtggaggttatcaaagaatctcttggtgaagaggtttttaaaatatgttacgaggaa<br>gatgaaaacatccttgggggtggttggaggcaccccttaaagatttttttaaacagcttcagtacccttctgaaacag<br>agcagccattgccaagaagcaggaaaaggggcaggcttgaggacgcctccattctatgcctggataagga<br>ggatgattttctacatgtttactacttcttccctaagagaaccacctccctgattcttcccggcatcataaaggcag<br>ctgctcacgtattatatgaaacggaagtggaagtgtcgttaatgcctccctgcttccataatgattgcagcgagtt<br>tgtgaatcagccctacttgttgtactccgttcacatgaaaagcaccaagccatccctgtcccccagcaaacccc<br>agtcctcgctggtgattcccacatcgctattctgcaagacatttccattccatttcatgtttgacaaagatatgaca<br>attctgcaatttggcaatggcatcagaaggctgatgaacaggagagactttcaaggaaagcctaattttgaaga<br>atactttgaaattctgactccaaaaatcaaccagacgtttagcgggatcatgactatgttgaatatgcagtttgttg<br>tacgagtgaggagatgggacaactctgtgaaaaaatcttcaagggttatggacctcaaaggccaaatgatcta<br>cattgttgaatccagtgcaatcttgtttttggggtcaccctgtgtggacagattagaagattttacaggacgaggg

	ctctacctctcagacatcccaattcacaatgcactgagggatgtggtcttaataggggaacaagcccgagctca<br>agatggcctgaagaagaggctggggaagctgaaggctacccttgagcaagcccaccaagccctggagga<br>ggagaagaaaaagacagtagaccttctgtgctccatatttccctgtgaggttgctcagcagctgtggcaaggg<br>caagttgtgcaagccaagaagttcagtaatgtcaccatgctcttctcagacatcgttgggttcactgccatctgct<br>cccagtgctcaccgctgcaggtcatcaccatgctcaatgcactgtacactcgcttcgaccagcagtgtggaga<br>gctggatgtctacaaggtggagaccattggcgatgcctattgtgtagctgggggattacacaaagagagtgat<br>actcatgctgttcagatagcgctgatggccctgaagatgatggagctctctgatgaagttatgtctccccatgga<br>gaacctatcaagatgcgaattggactgcactctggatcagtttttgctggcgtcgttggagttaaaatgccccgtt<br>actgtctttttggaaacaatgtcactctggctaacaaatttgagtcctgcagtgtaccacgaaaaatcaatgtcag<br>cccaacaacttacagattactcaaagactgtcctggtttcgtgtttacccctcgatcaagggaggaacttccacc<br>aaacttccctagtgaaatccccggaatctgccattttctggatgcttaccaacaaggaacaaactcaaaaccatg<br>cttccaaaagaaagatgtggaagatggcaatgccaatttttaggcaaagcatcaggaatagattag
guanylate cyclase 1<br>soluble subunit<br>beta 1 (GUCY1B1)	atgtacggatttgtgaatcacgccctggagttgctggtgatccgcaattacggccccgaggtgtgggaagaca<br>tcaaaaaagaggcacagttagatgaagaaggacagtttcttgtcagaataatatatgatgactccaaaacttatg<br>atttggttgctgctgcaagcaaagtcctcaatctcaatgctggagaaatcctccaaatgtttgggaagatgtttttc<br>gtcttttgccaagaatctggttatgatacaatcttgcgtgtcctgggctctaatgtcagagaatttctacagaacctt<br>gatgctctgcacgaccaccttgctaccatctacccaggaatgcgtgcaccttcctttaggtgcactgatgcaga<br>aaagggcaaaggactcattttgcactactactcagagagagaaggacttcaggatattgtcattggaatcatca<br>aaacagtggcacaacaaatccatggcactgaaatagacatgaaggttattcagcaaagaaatgaagaatgtg<br>atcatactcaatttttaattgaagaaaaagagtcaaaagaagaggatttttatgaagatcttgacagatttgaaga<br>aaatggtacccaggaatcacgcatcagcccatatacattctgcaaagcttttccttttcatataatatttgaccggg<br>acctagtggtcactcagtgtggcaatgctatatacagagttctcccccagctccagcctgggaattgcagccttc<br>tgtctgtcttctcgctggttcgtcctcatattgatattagtttccatgggatcctttctcacatcaatactgtttttgtatt<br>gagaagcaaggaaggattgttggatgtggagaaattagaatgtgaggatgaactgactgggactgagatcag<br>ctgcttacgtctcaagggtcaaatgatctacttacctgaagcagatagcatactttttctatgttcaccaagtgtcat<br>gaacctggacgatttgacaaggagagggctgtatctaagtgacatccctctgcatgatgccacgcgcgatctt<br>gttcttttgggagaacaatttagagaggaatacaaactcacccaagaactggaaatcctcactgacaggctaca<br>gctcacgttaagagccctggaagatgaaaagaaaaagacagacacattgctgtattctgtccttcctccgtctgt<br>tgccaatgagctgcggcacaagcgtccagtgcctgccaaaagatatgacaatgtgaccatcctctttagtggc<br>attgtgggcttcaatgctttctgtagcaagcatgcatctggagaaggagccatgaagatcgtcaacctcctcaa<br>cgacctctacaccagatttgacacactgactgattcccggaaaaacccatttgtttataaggtggagactgttgg<br>tgacaagtatatgacagtgagtggtttaccagagccatgcattcaccatgcacgatccatctgccacctggcctt<br>ggacatgatggaaattgctggccaggttcaagtagatggtgaatctgttcagataacaatagggatacacactg<br>gagaggtagttacaggtgtcataggacagcggatgcctcgatactgtctttttgggaatactgtcaacctcacaa<br>gccgaacagaaaccacaggagaaaagggaaaaataaatgtgtctgaatatacatacagatgtcttatgtctcc<br>agaaaattcagatccacaattccacttggagcacagaggcccagtgtccatgaagggcaaaaaagaaccaat<br>gcaagtttggtttctatccagaaaaaatacaggaacagaggaaacaaagcaggatgatgactag
GUCY1A1-P2A-GUCY1B1	atgttctgcacgaagctcaaggatctcaagatcacaggagagtgtcctttctccttactggcaccaggtcaagttcc<br>gagtcttcagaggaggcagcaggaagctcagagagctgcaaagcaaccgtgcccatctgtcaagacattcctga<br>gaacatacaagaaagtcttcctcaaagaaaaaccagtcggagccgagtctatcttcacactttggcagagagtatt<br>aactgattttcccagagtttgaacggctgaatgttgcacttcagagaacattggcaaagcacaaaataaaagaaag<br>gaaatctttggaaagagaagactttgaaaaaacaattgcagagcaagcagttgcagcaggagttccagtggagg<br>caaagaatctcttggtgaagaggtttttaaaatatgttacgaggaagatgaaaacatccttggggtggttggaggca<br>ttaaagatttttaaacagcttcagtacccttctgaaacagagcagccattgccaagaagcaggaaaaagggcag<br>tgaggacgcctccattctatgcctggataaggaggatgattttctacatgtttactacttcttccctaagagaaccacc<br>ctgattcttcccggcatcataaaggcagctgctcacgtattatatgaaacggaagtggaagtgtcgttaatgcctcc<br>ttccataatgattgcagcgagtttgtgaatcagccctacttgttgtactccgttcacatgaaaagcaccaagccatcc<br>cccccagcaaaccccagtcctcgctggtgattcccacatcgctattctgcaagacatttccattccatttcatgtttga<br>agatatgacaattctgcaatttggcaatggcatcagaaggctgatgaacaggagagactttcaaggaaagcctaa<br>aagaatactttgaaattctgactccaaaaatcaaccagacgtttagcgggatcatgactatgttgaatatgcagtttg<br>cgagtgaggagatgggacaactctgtgaaaaaatcttcaagggttatggacctcaaaggccaaatgatctacatt<br>aatccagtgcaatcttgtttttggggtcaccctgtgtggacagattagaagattttacaggacgagggctctacctct<br>acatcccaattcacaatgcactgagggatgtggtcttaataggggaacaagcccgagctcaagatggcctgaag

	aggctggggaagctgaaggctacccttgagcaagcccaccaagccctggaggaggagaagaaaaagacagt<br>ccttctgtgctccatatttccctgtgaggttgctcagcagctgtggcaagggcaagttgtgcaagccaagaagttc<br>atgtcaccatgctcttctcagacatcgttgggttcactgccatctgctcccagtgctcaccgctgcaggtcatcacc<br>tcaatgcactgtacactcgcttcgaccagcagtgtggagagctggatgtctacaaggtggagaccattggcgatg<br>ttgtgtagctgggggattacacaaagagagtgatactcatgctgttcagatagcgctgatggccctgaagatgatg<br>ctctctgatgaagttatgtctccccatggagaacctatcaagatgcgaattggactgcactctggatcagtttttgct<br>tcgttggagttaaaatgccccgttactgtctttttggaaacaatgtcactctggctaacaaatttgagtcctgcagtgt<br>cgaaaaatcaatgtcagcccaacaacttacagattactcaaagactgtcctggtttcgtgtttacccctcgatcaag<br>ggaacttccaccaaacttccctagtgaaatccccggaatctgccattttctggatgcttaccaacaaggaacaaac<br>aaccatgcttccaaaagaaagatgtggaagatggcaatgccaatttttaggcaaagcatcaggaatagataagc<br>cactaactttagtcttctgaaacaggccggtgatgtggaagagaatcccggccctatgtatgggttcgtgaacca<br>ttgagttactcgtgatccggaactacgggcctgaggtctgggaggacatcaagaaagaagcacagctggacga<br>ggggcagtttctggtccgcattatctatgacgacagcaagacctacgatctggttgcagccgcctctaaggtgct<br>ctgaatgctggtgagatcctgcaaatgttcgggaagatgttcttcgtgttctgtcaagagtctggctatgatacaatt<br>gggtcctgggcagtaacgtacgcgaattccttcagaacctggatgcactgcacgatcacctcgccaccatatac<br>ggatgcgagcccctagctttcggtgcacagatgccgaaaaaggaaagggcctgattctgcactattactccgaa<br>aaggcctgcaagatatcgtgatcggtataattaaaaccgtcgctcagcagatccatggtacagaaatcgacatga<br>gatccagcagcgtaacgaggagtgcgaccatacccagtttctgatagaagagaaggaatcaaaggaagaaga<br>acgaagacttggataggttcgaggaaaatggcactcaggaaagccgcatctccccatataccttctgtaaggcct<br>gttccatatcatttttgatagggacctcgtggtcacccagtgcggaaacgcaatttatagagtgctcccccaactgc<br>ccggcaattgtagcctattgagtgttttcagcctggtgaggcctcatatcgatatttcttttcacggaattttgagcca<br>aatactgtgttcgtcttgagatccaaagagggcctcctagacgtggaaaagcttgaatgtgaggacgagcttaca<br>cagaaatcagctgtctcagattgaaaggacagatgatctatttaccagaggctgactcaattctctttttatgttcgc<br>gttatgaatctcgacgatttgaccagacgcggcctgtacctgtcagacattccccttcatgatgctacccgggactt<br>ttactcggcgagcaattccgggaggagtacaagctgactcaggagttggaaatcctaacggatcgactgcaact<br>ctgcgagcactcgaagatgaaaagaaaaagacagacaccctcctatactcggtgcttccaccatcagtagctaa<br>ctaaggcacaagcgtcccgtaccggcaaaaagatatgacaacgtgacaattctttttcaggcatagttggcttca<br>cttttgtagcaaacacgcttccggagagggggcgatgaaaattgttaacctgctgaatgacctgtatacacgattt<br>cccttactgacagtcgtaaaaatccctttgtttacaaggtggagacggtgggagataagtacatgacggtgtctgg<br>cccgagccatgcattcatcacgcgagatccatctgccacttggctctggatatgatggaaatcgccggccaggt<br>gtggacggggagtctgtacagatcaccatcggtattcataccggagaagtcgtcacaggagtcatcggacaga<br>gccacgctactgcctgttcggtaacacggtaaacctaacatcccggactgagacaactggggagaaaggcaag<br>acgtttccgagtacacctaccgctgcctcatgagcccagagaactctgacccgcagtttcatctcgagcaccggg<br>tgtctccatgaaaggaaagaaggagcctatgcaggtctggtttctcagtagaaagaatactgggaccgaggaga<br>gcaggatgatgactag
P_{CRE}-NLuc	gcaccagacagtgacgtcagctgccagatcccatggccgtcatactgtgacgtctttcagacaccccattgac<br>gtcaatgggagaacagatctgccgccccgactgcatctgcgtgttcgaattcgccaatgacaagacgctggg<br>cggggtttgtgtcatcatagaactaaagacatgcaaatatatttcttccggggacaccgccagcaaacgcgag<br>caacgggccacggggatgaagcaccatgactagtgagacagacacactcctgctatgggtactgctgctctg<br>ggttccaggttccactggtgacgctagtggtggttctggtatggtcttcacactcgaagatttcgttggggactg<br>gcgacagacagccggctacaacctggaccaagtccttgaacagggaggtgtgtccagtttgtttcagaatctc<br>ggggtgtccgtaactccgatccaaaggattgtcctgagcggtgaaaatgggctgaagatcgacatccatgtca<br>tcatcccgtatgaaggtctgagcggcgaccaaatgggccagatcgaaaaaatttttaaggtggtgtaccctgtg<br>gatgatcatcactttaaggtgatcctgcactatggcacactggtaatcgacggggttacgccgaacatgatcga<br>ctatttcggacggccgtatgaaggcatcgccgtgttcgacggcaaaaagatcactgtaacagggaccctgtg<br>gaacggcaacaaaattatcgacgagcgcctgatcaaccccgacggctccctgctgttccgagtaaccatcaa<br>cggagtgaccggctggcggctgtgcgaacgcattctggcggactacaaagaccatgacggtgattataaag<br>atcatgacatcgattacaaggatgacgatgacaagtag
aldehyde dehydrogenase 2 family member (ALDH2)	atgttgcgcgctgccgcccgcttcgggccccgcctgggccgccgcctcttgtcagccgccgccacccaggc<br>cgtgcctgcccccaaccagcagcccgaggtcttctgcaaccagattttcataaacaatgaatggcacgatgcc<br>gtcagcaggaaaacattccccaccgtcaatccgtccactggagaggtcatctgtcaggtagctgaaggggac<br>aaggaagatgtggacaaggcagtgaaggccgcccgggccgccttccagctgggctcaccttggcgccgca<br>tggacgcatcacacaggggccggctgctgaaccgcctggccgatctgatcgagcgggaccggacctacct

	ggcggccttggagaccctggacaatggcaagccctatgtcatctcctacctggtggatttggacatggtcctca<br>aatgtctccggtattatgccggctgggctgataagtaccacgggaaaaccatccccattgacggagacttcttc<br>agctacacacgccatgaacctgtgggggtgtgcgggcagatcattccgtggaatttcccgctcctgatgcaag<br>catggaagctgggcccagccttggcaactggaaacgtggttgtgatgaaggtagctgagcagacacccctca<br>ccgccctctatgtggccaacctgatcaaggaggctggctttccccctggtgtggtcaacattgtgcctggatttg<br>gccccacggctggggccgccattgcctcccatgaggatgtggacaaagtggcattcacaggctccactgag<br>attggccgcgtaatccaggttgctgctgggagcagcaacctcaagagagtgaccttggagctggggggaa<br>gagccccaacatcatcatgtcagatgccgatatggattgggccgtggaacaggcccacttcgccctgttcttca<br>accagggccagtgctgctgtgccggctcccggaccttcgtgcaggaggacatctatgatgagtttgtggagc<br>ggagcgttgcccgggccaagtctcgggtggtcgggaacccctttgatagcaagaccgagcaggggccgca<br>ggtggatgaaactcagtttaagaagatcctcggctacatcaacacggggaagcaagagggggcgaagctgc<br>tgtgtggtgggggcattgctgctgaccgtggttacttcatccagcccactgtgtttggagatgtgcaggatggc<br>atgaccatcgccaaggaggagatcttcgggccagtgatgcagatcctgaagttcaagaccatagaggaggtt<br>gttgggagagccaacaattccacgtacgggctggccgcagctgtcttcacaaaggatttggacaaggccaatt<br>acctgtcccaggccctccaggcgggcactgtgtgggtcaactgctatgatgtgtttggagcccagtcaccctttt<br>ggtggctacaagatgtcggggagtggccgggagttgggcgagtacgggctgcaggcatacactgaagtga<br>aaactgtcacagtcaaagtgcctcagaagaactcataa
protein kinase cGMP-dependent 1 (PRKG1)	atggaattcggcaccttgcgggatttacagtacgcgctccaggagaagatcgaggagctgaggcagcggga<br>tgctctcatcgacgagctggagctggagttggatcagaaggacgaactgatccagaagctgcagaacgagct<br>ggacaagtaccgctcggtgatccgaccagccacccagcaggcgcagaagcagagcgcgagcaccttgca<br>gggcgagccgcgcaccaagcggcaggcgatctccgccgagcccaccgccttcgacatccaggatctcagc<br>catgtgaccctgcccttctaccccaagagcccacagtccaaggatcttataaaggaagctatccttgacaatga<br>ctttatgaagaacttggagctgtcgcagatccaggagattgtggattgtatgtacccggtggagtatggcaagg<br>acagttgcatcatcaaagaaggagacgtggggtcactggtgtatgtcatggaagatggtaaggttgaagttac<br>aaaagaaggtgtgaagttgtgtaccatgggtccaggaaaagtgtttggggaattggctattctttacaactgtac<br>ccggacagcgaccgtcaagactcttgtaaatgtaaaactctgggccattgatcgacaatgttttcaaacaataat<br>gatgaggacaggactcatcaagcataccgagtatatggaatttttaaaaagcgttccaacattccagagccttc<br>ctgaagagatcctcagcaagcttgctgatgtccttgaagagacccactatgaaaatggagaatatattatcagg<br>caaggtgcaagaggggacaccttctttatcatcagcaaaggaacggtaaatgtcactcgtgaagactcaccga<br>gtgaagacccagtctttcttagaactttaggaaaaggagactggtttggagagaaagccttgcaggggggaaga<br>tgtgagaacagcaaacgtaattgctgcagaagctgtaacctgccttgtgattgacagagactcttttaaacatttg<br>attggagggctggatgatgtttctaataaagcatatgaagatgcagaagctaaagcaaaatatgaagctgaag<br>cggctttcttcgccaacctgaagctgtctgatttcaacatcattgataccctttggagttggaggtttcggacgagt<br>agaactggtccagttgaaaagtgaagaatccaaaacgtttgcaatgaagattctcaagaaacgtcacattgtgg<br>acacaagacagcaggaacacatccgctcagagaagcagatcatgcaggggggctcattccgatttcatagtga<br>gactgtacagaacatttaaggacagcaaatatttgtatatgttgatggaagcttgtctaggtggagagctctgga<br>ccattctcagggatagaggttcgtttgaagattctacaaccagattttacacagcatgtgtggtagaagcttttgc<br>ctatctgcattccaaaggaatcatttacagggacctcaagccagaaaatctcatcctagatcaccgaggttatgc<br>caaactggttgattttggctttgcaaagaaaataggatttggaaagaaaacatggactttttgtgggactccaga<br>gtatgtagccccagagatcatcctgaacaaaggccatgacgtttcagccgactactggtcactgggaatccta<br>atgtatgaactcctgactggcagcccacctttctcaggcccagatcctatgaaaacctataacatcatattgagg<br>gggattgacatgatagaatttccaaagaagattgccaaaaatgctgctaatttaattaaaaaactatgcagggac<br>aatccatcagaaagattagggaatttgaaaaatggagtaaaagacattcaaaagcacaaatggtttgagggctt<br>taactgggaaggcttaagaaaaggtaccttgacacctcctataataccaagtgttgcatcacccacagacacaa<br>gtaattttgacagtttccctgaggacaacgatgaaccaccacctgatgacaactcaggatgggatatagacttc<br>gaattctatccttacgacgtgcctgactacgcctaa
P_{CRE}-GLP-1-Fc-P2A-NLuc	gcaccagacagtgacgtcagctgccagatcccatggccgtcatactgtgacgtctttcagacaccccattgacgt<br>gggagaacagatctgccgccccgactgcatctgcgtgttcgaattcgccaatgacaagacgctgggcggggtt<br>catcatagaactaaagacatgcaaatatatttcttccggggacaccgccagcaaacgcgagcaacgggccacg<br>tgaagcaccatgactagtaagatcatcctgtggctgtgtgtgttcggcctgttcctggccaccctgttccccatcag<br>cagatgcccgtggagtccggcctgtcctccgaggactccgccagctccgagagcttcgccaagcgcatcaag

	acggcgagggcaccttcaccagcgacgtgagcagctacctggagggccaggccgccaaggagttcatcgcc<br>tggtgaagggccgcggcagatctggttgtaagccttgcatatgtacagtcccagaagtatcatctgtcttcatcttc<br>caaagcccaaggatgtgctcaccattactctgactcctaaggtcacgtgtgttgtggtagacatcagcaaggatg<br>gaggtccagttcagctggtttgtagatgatgtggaggtgcacacagctcagacgcaaccccgggaggagcagt<br>cagcactttccgctcagtcagtgaacttcccatcatgcaccaggactggctcaatggcaaggagttcaaatgcag<br>aacagtgcagctttccctgcccccatcgagaaaaccatctccaaaaccaaaggcagaccgaaggctccacagg<br>caccattccacctcccaaggagcagatggccaaggataaagtcagtctgacctgcatgataacagacttcttccc<br>gacattactgtggagtggcagtggaatgggcagccagcggagaactacaagaacactcagcccatcatggac<br>atggctcttacttcgtctacagcaagctcaatgtgcagaagagcaactgggaggcaggaaatactttcacctgct<br>ttacatgagggcctgcacaaccaccatactgagaagagcctctcccactctcctggtaaaggaagcggagcta<br>ttcagcctgctgaagcaggctggagacgtggaggagaaccctggaccttccggagagacagacacactcctg<br>ggtactgctgctctgggttccaggttccactggtgacgctggttctggtggttctatggtcttcacactcgaagatt<br>gggactggcgacagacagccggctacaacctggaccaagtccttgaacagggaggtgtgtccagtttgtttcag<br>tcggggtgtccgtaactccgatccaaaggattgtcctgagcggtgaaaatgggctgaagatcgacatccatgtc<br>cccgtatgaaggtctgagcggcgaccaaatgggccagatcgaaaaaatttttaaggtggtgtaccctgtggatg<br>cactttaaggtgatcctgcactatggcacactggtaatcgacggggttacgccgaacatgatcgactatttcgga<br>cgtatgaaggcatcgccgtgttcgacggcaaaaagatcactgtaacagggaccctgtggaacggcaacaaaa<br>gacgagcgcctgatcaaccccgacggctccctgctgttccgagtaaccatcaacggagtgaccggctggcgg<br>cgaacgcattctggcgtag

Supplementary References

1. Haellman, V., Strittmatter, T., Bertschi, A., Stücheli, P. & Fussenegger, M. A versatile plasmid architecture for mammalian synthetic biology (VAMSyB). *Metab Eng* **66**, 41-50 (2021).
2. Mahameed, M., Xue, S., Stefanov, B.A., Hamri, G.C. & Fussenegger, M. Engineering a Rapid Insulin Release System Controlled By Oral Drug Administration. *Adv Sci (Weinh)* **9**, e2105619 (2022).
3. Xue, S. et al. A Synthetic-Biology-Inspired Therapeutic Strategy for Targeting and Treating Hepatogenous Diabetes. *Mol Ther* **25**, 443-455 (2017).
4. Deguchi, A. et al. Vasodilator-stimulated phosphoprotein (VASP) phosphorylation provides a biomarker for the action of exisulind and related agents that activate protein kinase G. *Mol Cancer Ther* **1**, 803-809 (2002).
5. Johannessen, C.M. et al. COT drives resistance to RAF inhibition through MAP kinase pathway reactivation. *Nature* **468**, 968-972 (2010).
6. Kemmer, C. et al. A designer network coordinating bovine artificial insemination by ovulation-triggered release of implanted sperms. *J Control Release* **150**, 23-29 (2011).
7. Bai, P. et al. A fully human transgene switch to regulate therapeutic protein production by cooling sensation. *Nat Med* **25**, 1266-1273 (2019).
8. Huang, J., Xue, S., Buchmann, P., Teixeira, A.P. & Fussenegger, M. An electrogenetic interface to program mammalian gene expression by direct current. *Nat Metab* **5**, 1395-1407 (2023).
9. Gitzinger, M., Kemmer, C., El-Baba, M.D., Weber, W. & Fussenegger, M. Controlling transgene expression in subcutaneous implants using a skin lotion containing the apple metabolite phloretin. *Proc Natl Acad Sci U S A* **106**, 10638-10643 (2009).
10. Xie, M. et al. β-cell-mimetic designer cells provide closed-loop glycemic control. *Science* **354**, 1296-1301 (2016).
