# Supplementary material for: Nitroglycerin-responsive gene switch for the on-demand production of therapeutic proteins
Source: Nat Biomed Eng. 2025 Feb 14;9(7):1129–43. doi: 10.1038/s41551-025-01350-7 (PMC12270905; doi:10.1038/s41551-025-01350-7)
Supplement: Supplementary file 2 — Reporting Summary [file 41551_2025_1350_MOESM2_ESM.pdf]

nature portfolio

Corresponding author(s): Martin Fussenegger

Last updated by author(s): Nov 18, 2024

Reporting Summary

Nature Portfolio wishes to improve the reproducibility of the work that we publish. This form provides structure for consistency and transparency in reporting. For further information on Nature Portfolio policies, see our Editorial Policies and the Editorial Policy Checklist.

Statistics

For all statistical analyses, confirm that the following items are present in the figure legend, table legend, main text, or Methods section.

n/a	Confirmed	
☐	☒	The exact sample size (*n*) for each experimental group/condition, given as a discrete number and unit of measurement
☐	☒	A statement on whether measurements were taken from distinct samples or whether the same sample was measured repeatedly
☐	☒	The statistical test(s) used AND whether they are one- or two-sided<br>*Only common tests should be described solely by name; describe more complex techniques in the Methods section.*
☒	☐	A description of all covariates tested
☐	☒	A description of any assumptions or corrections, such as tests of normality and adjustment for multiple comparisons
☐	☒	A full description of the statistical parameters including central tendency (e.g. means) or other basic estimates (e.g. regression coefficient) AND variation (e.g. standard deviation) or associated estimates of uncertainty (e.g. confidence intervals)
☐	☒	For null hypothesis testing, the test statistic (e.g. F, t, r) with confidence intervals, effect sizes, degrees of freedom and P value noted<br>*Give P values as exact values whenever suitable.*
☒	☐	For Bayesian analysis, information on the choice of priors and Markov chain Monte Carlo settings
☒	☐	For hierarchical and complex designs, identification of the appropriate level for tests and full reporting of outcomes
☒	☐	Estimates of effect sizes (e.g. Cohen's *d*, Pearson's *r*), indicating how they were calculated

Our web collection on statistics for biologists contains articles on many of the points above.

Software and code

Policy information about availability of computer code

Data collection	Absorbance, luminescence, and fluorescence data were collected using TECAN AG, Maennedorf, Switzerland. Western blot images were developed using FUSION Pulse TS (cat. no.37480003, Vilber, France). Blood glucose concentrations measured by a clinically licensed glucometer (Accu-Check Instant, Roche). Blood pressure and heart rate were analyzed using a computerized, non-invasive tail-cuff system (Bioseb, France, cat. no. BP-2000). RNA libraries were pooled and sequenced SR101 on an Illumina NovaSeq 6000 system.
Data analysis	GraphPad Prism 9 and adobe illustrator 2021. Statistical analysis of deep sequencing was performed with R scripts using edgeR (v4.0.16).

For manuscripts utilizing custom algorithms or software that are central to the research but not yet described in published literature, software must be made available to editors and reviewers. We strongly encourage code deposition in a community repository (e.g. GitHub). See the Nature Portfolio guidelines for submitting code & software for further information.

Data

Policy information about availability of data

All manuscripts must include a data availability statement. This statement should provide the following information, where applicable:

- Accession codes, unique identifiers, or web links for publicly available datasets
- A description of any restrictions on data availability
- For clinical datasets or third party data, please ensure that the statement adheres to our policy

The main data supporting the results in this study are available within the paper and its Supplementary Information. Source data for the figures are provided with this paper. All raw and analysed data generated during the study are available from the corresponding author on reasonable request.

Research involving human participants, their data, or biological material

Policy information about studies with human participants or human data. See also policy information about sex, gender (identity/presentation), and sexual orientation and race, ethnicity and racism.

Reporting on sex and gender	The study did not involve human research participants.
Reporting on race, ethnicity, or other socially relevant groupings	–
Population characteristics	–
Recruitment	–
Ethics oversight	–

Note that full information on the approval of the study protocol must also be provided in the manuscript.

Field-specific reporting

Please select the one below that is the best fit for your research. If you are not sure, read the appropriate sections before making your selection.

☒ Life sciences ☐ Behavioural & social sciences ☐ Ecological, evolutionary & environmental sciences

For a reference copy of the document with all sections, see nature.com/documents/nr-reporting-summary-flat.pdf

Life sciences study design

All studies must disclose on these points even when the disclosure is negative.

Sample size	No statistical methods were used to predetermine sample size. However, based on our previous experiments and the existing literature with similar settings, when we used the same sample number of control and treated group and assuming type-I error = 0.05 and the probability of a type-II error = 0.20, we required at least 2.6 samples for each group to detect a 50% change between groups means. Therefore, we used at least 3 replicates for each group (Bai et al., Nat Med., 2019).
Data exclusions	No data were excluded.
Replication	All experiments were successfully repeated at least three times.
Randomization	Samples were randomly allocated into different experimental groups. For each mouse study, animals of the same genetic background were randomly allocated into different experimental groups.
Blinding	The investigators were not blinded to allocation during the experiments and outcome assessment. Blinding was not possible, as the same investigator processed the experiments and analysed the data.

Reporting for specific materials, systems and methods

We require information from authors about some types of materials, experimental systems and methods used in many studies. Here, indicate whether each material, system or method listed is relevant to your study. If you are not sure if a list item applies to your research, read the appropriate section before selecting a response.

Materials & experimental systems

n/a	Involved in the study	
☐	☒	Antibodies
☐	☒	Eukaryotic cell lines
☒	☐	Palaeontology and archaeology
☐	☒	Animals and other organisms
☒	☐	Clinical data
☒	☐	Dual use research of concern
☒	☐	Plants

Methods

n/a	Involved in the study	
☒	☐	ChIP-seq
☒	☐	Flow cytometry
☒	☐	MRI-based neuroimaging

Antibodies

Antibodies used	The following primary antibodies were used in this study (all in 1:1000 dilution): monoclonal anti-ALDH2 antibody produced in rabbit (Cell Signaling, cat. no. 18818) and mouse anti-α-actinin (Cell Signaling, cat. no. 69758, clone E7U1O). Secondary HRP-conjugated goat anti-rabbit (cat. no. 111-035-144, polyclonal) and anti-mouse (cat. no. 115-035-003, polyclonal) antibodies were purchased from Jackson Immunoresearch, West Grove, PA, and used at a dilution of 1:10,000 dilution.<br>For ICH, we used leaved Caspase 3 antibody (Cell Signaling Technology, Lot Nr.: 47, Catalog Nr.: 9661S, in 1:500 dilution)
Validation	Anti-ALDH2 antibody was validated by transfection of HEK-293 cells with human ALHD2 encoding expression vector followed by immunoblotting.<br>Mouse anti-α-actinin was validated by loading different amounts of cell lysate. A corelation between the lysate amount and band intensity in the correct protein size was noticed.<br>Both secondary antibodies were validated by the absence of bands/signal in immunoblot that has not been exposed to primary antibody. Cleaved Caspase-3 antibody was validated by specific staining of a control human tonsil and rat spleen. The technical negative controls showed no staining in the capsules that could be attributed to unspecific binding of the used antibody.

Eukaryotic cell lines

Policy information about cell lines and Sex and Gender in Research

Cell line source(s)	Human embryonic kidney cells (HEK-293T, ATCC: CRL-11268), HeLa cells<br>(HeLa, ATCC: CCL-2), HepG2 cells (HEPG2, ATCC: HB-8065), baby hamster kidney cells (BHK, ATCC: CCL-10), and HT-1080 cells (HT1080, ATCC: CCL-121), Chinese hamster ovary cells (CHOK1, ATCC: CCL-61), A549 cells (A549, ATCC: CCL-185), and Primary dermal fibroblast normal; human, neonatal (HDFn; ATCC, PCS-201-010).
Authentication	Cells were authenticated by ATCC. All the phenotypes of cell lines were frequently checked and controlled by microscopy.
Mycoplasma contamination	Cells were tested frequently for mycoplasma, and confirmed as negative.
Commonly misidentified lines<br>(See ICLAC register)	No commonly misidentified cell lines were used.

Animals and other research organisms

Policy information about studies involving animals; ARRIVE guidelines recommended for reporting animal research, and Sex and Gender in Research

Laboratory animals	12-week-old male C57BL/6 or db/db mice (BKS.Cg-Dock7m +/+ Leprdb/J, derived from C57BL/6J, Janvier Labs Saint-Berthevin, France) were used.
Wild animals	The study did not involve wild animals.
Reporting on sex	Male mice were used, due to husbandry convenience, as supported by: Bai et al., Nature Medicine,2019; Krawczyk et al., Science, 2020; Chen et al, Nature Chemical Biology; and Zhou et al., Nature Biotechnology, 2021. Sex was not considered in the study design. No data disaggregated for sex were collected.
Field-collected samples	The study did not involve samples collected from the field.
Ethics oversight	All animal experiments were conducted in compliance with the directive of the European Community Council (2010/63/EU) and approved by the French Republic (project no. DR2013-v2, licenses: Ghislaine Charpin-El Hamri, no. 69266309, and Shuai Xue, no. LTK4899). In addition, experiments were also performed following the Animal Care Guidelines of the Ministry of Science and Technology of the People's Republic of China and were approved by the Institutional Animal Care and Use Committee (IACUC) of Westlake University (Protocol ID: AP#24-088-XMQ). We adhered to rigorous ethical standards to ensure the welfare of the animals involved, and our study design was guided by the principles of the 3Rs: replacement, reduction and refinement.

Note that full information on the approval of the study protocol must also be provided in the manuscript.
